# Supplementary material for: Nymphal diets boost adults’ immunity via strengthened constitutive immunity and metabolic capacity in Adelphocoris suturalis
Source: Commun Biol. 2025 Dec 13;9:88. doi: 10.1038/s42003-025-09352-6 (PMC12820365; doi:10.1038/s42003-025-09352-6)
Supplement: Supplementary file 2 — Supplementary Information [file 42003_2025_9352_MOESM2_ESM.pdf]

Supplementary Table 1. Primers for quantitative real-time PCR analysis.

Gene	Direction	Sequence（5'->3'）	PCR efficiency (%)	Standard curve R^2
qAs-RPS15	Forward	GCGCCTCCAAATGAAAAGCCCG	92.6	0.997
	Reverse	GGGCCTTCCGTGTTTGACAGGT		
qAs-GNBP1e	Forward	GCTCGCATGCAAATGGTGGA	98.4	0.992
	Reverse	GAGATTGCTAAGGGGGCGGT		
qAs-CTL2	Forward	GCTGACAGGCCTCTAGGACG	103.1	0.996
	Reverse	CGGTCCCGCTGGTTTTCTTG		
qAs-SPZ	Forward	TGTTCGTCGTGGCCATCTAT	97.1	0.994
	Reverse	TTGTCCGAACGGCCAAACTT		
qAs-Toll4b	Forward	AAGCGCCGAAGGAATCTCGT	109.1	0.995
	Reverse	GGGTAAGGGAACACCTGCGA		
qAs-Tube2	Forward	ACCTTGCCAGTACCGACACC	99.2	0.998
	Reverse	CGCCAAGGAGGAGTTCTGGT		
qAs-Pelle1	Forward	GGGTCGATTTCCCGCCCAAT	105.7	0.995
	Reverse	GAACTTGGCAGAGGTGGCGA		
qAs-cSP16	Forward	CCGTTGTGTTCATGGCGCTT	99.5	0.998
	Reverse	ACCCCTACACACAGAGCGTG		
qAs-Lys	Forward	GACCACGGGCTCTTCCAAAT	97.9	0.997
	Reverse	TTCCCCTGTTGTCGTTTCGT		
qAs-Alo2a	Forward	CTGAGGAAGCGGTGACGAGT	102.6	0.997
	Reverse	ATGCCACCCAACAATTCGCC		
qAs-HK1	Forward	TGACGATTGCCGTGGATGGA	98.1	0.995
	Reverse	ATGCGATGCCTGCACCTTTG		

qAs-Glys59	Forward	ACGTCGGCTCCCTTGGTTAG	99	0.992
	Reverse	CCGACTGAGGGCCAAGTTCA		
qAs-TPS1	Forward	CGTGGCGGTGCTAAACCTTC	106.1	0.996
	Reverse	AAGGGCTTCCTCAGCGTCTC		
qAs-PFK	Forward	ATGCTGCCGACGGTGTGAT	97.8	0.992
	Reverse	TTGGGCTAAGAACACCTTCTCGG		
qAs-PK1	Forward	CGTCTGCCCCGACTTTCTTG	100.4	0.997
	Reverse	TGGAGGCAGGTCCAATCGTG		
qAs-IDH2	Forward	CCGTATCCCTGAGCAACCGA	104.9	0.995
	Reverse	ACCCGATGTACCTGAGCACG		
qAs-KGDH3	Forward	ACGCCCTTGTCTTGTGGGAA	99.8	0.997
	Reverse	CGGATGGCGAACTCCTCACT		
qAs-Scs2	Forward	TCGTTCCCTGAAGACGGCAA	99.4	0.995
	Reverse	GCGAGCCTGCCAATTTCCTG		
qAs-SDH1	Forward	ACAACGTCTCGGGAAGCCAA	98.7	0.996
	Reverse	TGAGGGCTGTCGTGGTGAAG		
qAs-FAS1b	Forward	ACCTTGGTCCCTGTTCCGTG	93.9	0.997
	Reverse	TGGCAACGGCTACGTCAGAA		
qAs-Acsl3	Forward	GGGAAGGTCGAAGCGGAGTT	96.8	0.995
	Reverse	CCAAGCGGTCTGCGATTTCC		
qAs-GAPDH1	Forward	CATTGACGGAGGCGCGAAAA	97.6	0.994
	Reverse	CTCCGACGACGAACATGGGA		
qAs-Lipase1f	Forward	GCGAAGAGGAAGTTGTCGCA	104.2	0.997
	Reverse	TCACCAACAGCCCTCTGCTC		
qAs-Gpat4a	Forward	GATCGGCGCGATCAAAACGA	97.5	0.998
	Reverse	GGTGCACGATTTCGGCCTTT		

qAs-HADH1	Forward	ATAATTGTAAAATCCCTCAC	98.7	0.998
	Reverse	TTTCCCGAGAACCCTCTCTTC		
qAs-GOGAT1d	Forward	GTTCGGTGGGTCAAGGACGA	101.3	0.998
	Reverse	AAGACTGACCACGACGGCAA		
qAs-ALT2	Forward	TCCCGCCCAATCAAAGGTGA	96.5	0.994
	Reverse	CTTGGCAGAGGTGGCGAGAT		
qAs-GS2a	Forward	GGGTCGGGGAAGATAGCGAC	98.4	0.998
	Reverse	GACCAGCTCCCCGAATGGAA		
qAs-GCL3	Forward	GCGGATATGGCGTGCTGAAG	98.3	0.998
	Reverse	GCGCATCGGACGATGATGTG		
qAs-PAH1	Forward	AGCACTTCCACGCTCTGTGT	102.6	0.995
	Reverse	CAGCACGCCATATCCGCTTC		
qAs-TH1	Forward	TGAGCATCCGTAGCGCGTAA	101.3	0.996
	Reverse	CTCACCTGGGAAGACGCACA		
qAs-PLOD	Forward	TACAGGCAGGCACGGTAGTG	105.6	0.993
	Reverse	TGATGGAGACCCACGCCAAG		

Supplementary Table 2. Summary of unigenes and transcripts statistics.

	Unigenes	Transcripts
Total number of sequences	96,293	152,451
Total base	103,043,820	164,149,189
Maximum length (bp)	35,349	35,349
Minimum length (bp)	201	201
Average length (bp)	1070.11	1076.73
Percent GC (%)	41.74	41.16

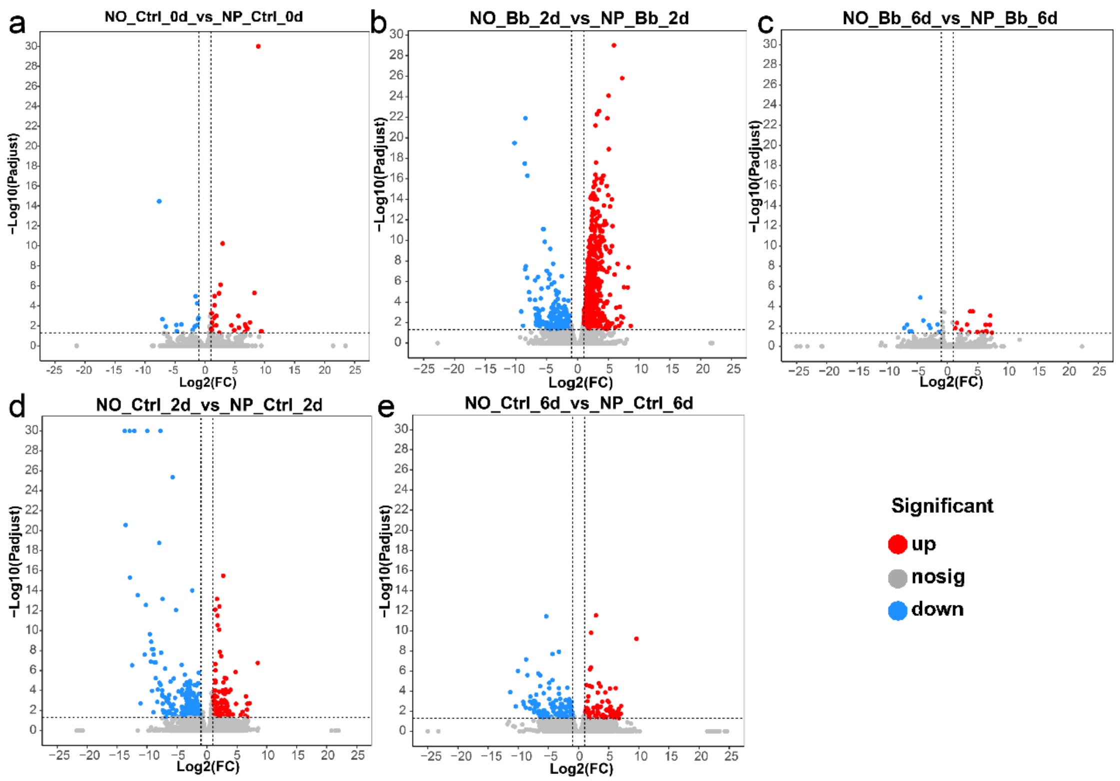

Supplementary Figure. 1 Volcano map of the differentially expressed immune-related genes between NP and NO groups. (a-c) Volcano plots showing differentially expressed genes (DEGs) at distinct time points under uninfected conditions. (d, e) Volcano plots showing DEGs at distinct time points under *Beauveria bassiana* infection. Upregulated genes are shown in red; downregulated genes are shown in blue. NO: adults developed from nymphs fed on an omnivorous diet; NP: adults developed from nymphs fed on a phytophagous diet; Bb: newly-emerged adults infected with *Beauveria bassiana*, Ctrl: newly-emerged adults treated with Tween 80.

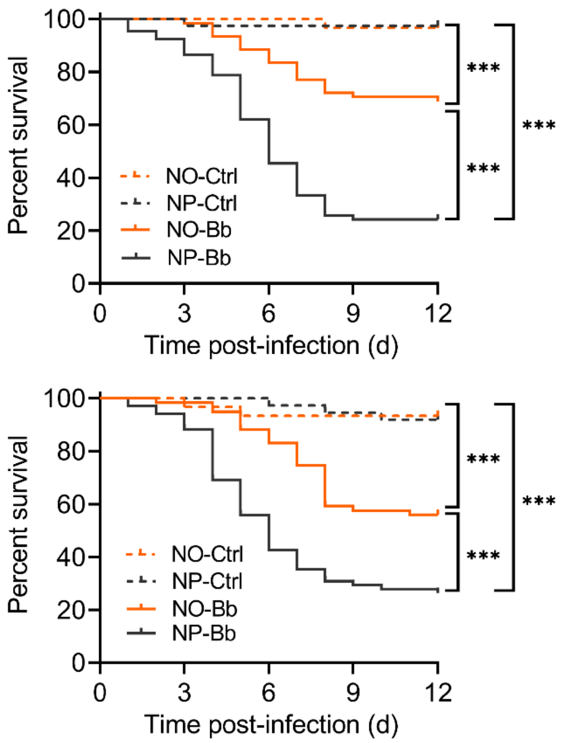

Supplementary Figure. 2 Replicate trials of *Adelphocoris Suturalis* adults' survival curves from different nymphal diets following *Beauveria Bassiana* infection. n = 30-61 biologically independent samples. NO: adults developed from nymphs fed on an omnivorous diet; NP: adults developed from nymphs fed on an herbivorous diet; Bb: newly-emerged adults infected with *Beauveria bassiana*, Ctrl: newly-emerged adults treated with Tween 80. Survival curves were monitored up to 12 days, while the observation window for statistical analysis was limited to 8 days, as mortality showed no substantial changes beyond this period. Log-rank tests were employed to compare and analyze the survival curves. (***$p < 0.001$).

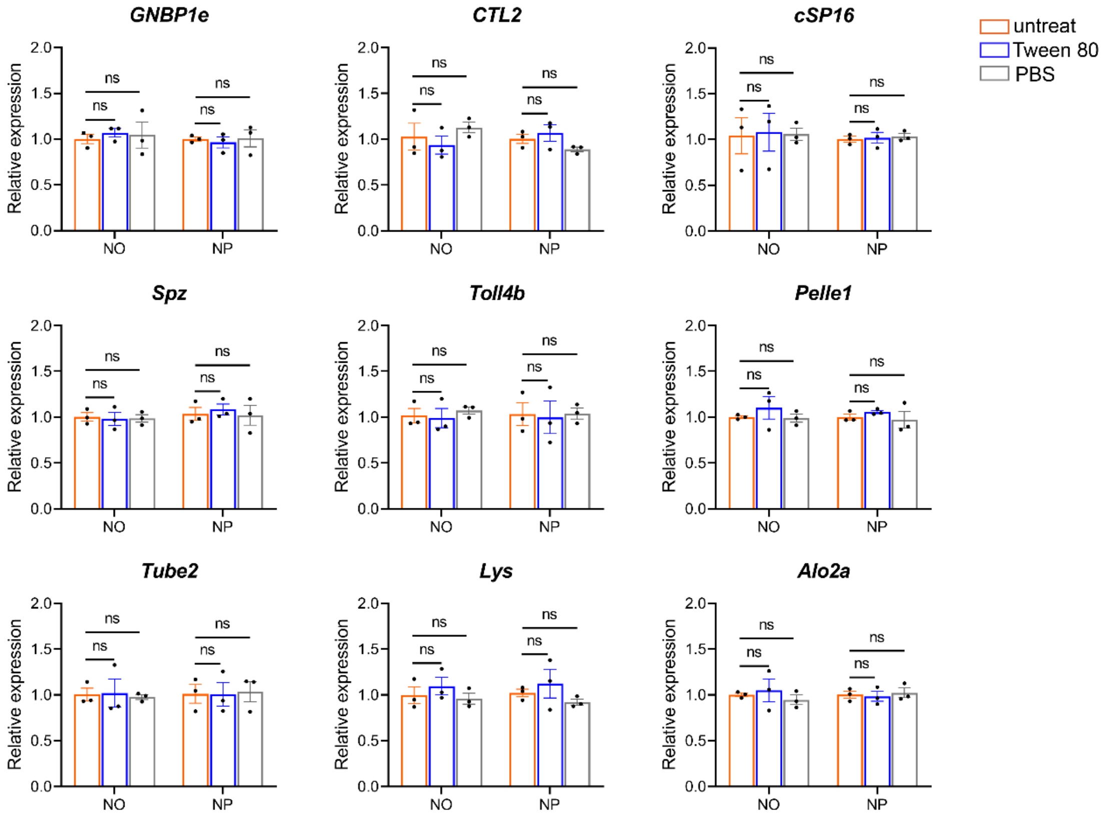

Supplementary Figure. 3 Effect of PBS and Tween 80 treatments on the expression of immune-related genes in *Adelphocoris suturalis* adults. n = 3 biologically independent samples. Relative expression levels of representative immune-related genes were measured by RT-qPCR. NO: adults developed from nymphs fed on an omnivorous die; NP: adults developed from nymphs fed on a phytophagous diet; untreat: newly-emerged adults with no treatment; Tween 80: newly-emerged adults treated with Tween 80; PBS: newly-emerged adults injected with PBS. Expression levels were calculated relative to the untreat group (set as 1.0) after normalization to the reference gene (*RPS15*). Statistical significance was determined using independent-sample t-test comparing Tween 80 vs.

untreat and PBS vs. untreat groups. The data are presented as means ± SEM. The significance of the differences (ns, not significant) was determined by the t-test.
