# Supplementary material for: Nymphal diets boost adults’ immunity via strengthened constitutive immunity and metabolic capacity in Adelphocoris suturalis
Source: Commun Biol. 2025 Dec 13;9:88. doi: 10.1038/s42003-025-09352-6 (PMC12820365; doi:10.1038/s42003-025-09352-6)
Supplement: Supplementary file 3 — Description of Additional Supplementary Files [file 42003_2025_9352_MOESM3_ESM.pdf]

Description of Additional Supplementary files

File name: Supplementary Data 1

Description: Results of the two-way analysis of variance (ANOVA) with Tukey's test for response of physiological immune parameters, macronutrient content and expression of candidate genes to diet and infection/injection.

File name: Supplementary Data 2

Description: Immune- and metabolic-related genes of *Adelphocoris suturalis* exposed to *Beauveria bassiana* for 0, 2 and 6 d in transcriptome.

File name: Supplementary Data 3

Description: The source data behind the graphs in the paper
